# A Genome Wide Association Study Revealed Key Single Nucleotide Polymorphisms/Genes Associated With Seed Germination in *Gossypium hirsutum* L.

**DOI:** 10.3389/fpls.2022.844946

**Published:** 2022-03-16

**Authors:** Aijun Si, Zhengwen Sun, Zhikun Li, Bin Chen, Qishen Gu, Yan Zhang, Liqiang Wu, Guiyin Zhang, Xingfen Wang, Zhiying Ma

**Affiliations:** ^1^State Key Laboratory of North China Crop Improvement and Regulation, North China Key Laboratory for Crop Germplasm Resources of Education Ministry, Key Laboratory for Crop Germplasm Resources of Hebei, Hebei Agricultural University, Baoding, China; ^2^Key Laboratory of China Northwestern Inland Region, Ministry of Agriculture, Cotton Research Institute, Xinjiang Academy of Agricultural and Reclamation Science, Shihezi, China

**Keywords:** cotton, seed germination, GWAS, SNP, candidate genes

## Abstract

Fast and uniform seed germination is essential to stabilize crop yields in agricultural production. It is important to understand the genetic basis of seed germination for improving the vigor of crop seeds. However, little is known about the genetic basis of seed vigor in cotton. In this study, we evaluated four seed germination-related traits of a core collection consisting of 419 cotton accessions, and performed a genome-wide association study (GWAS) to explore important loci associated with seed vigor using 3.66 million high-quality single nucleotide polymorphisms (SNPs). The results showed that four traits, including germination potential, germination rate, germination index, and vigor index, exhibited broad variations and high correlations. A total of 92 significantly associated SNPs located within or near 723 genes were identified for these traits, of which 13 SNPs could be detected in multiple traits. Among these candidate genes, 294 genes were expressed at seed germination stage. Further function validation of the two genes of higher expression showed that *Gh_A11G0176* encoding Hsp70-Hsp90 organizing protein negatively regulated *Arabidopsis* seed germination, while *Gh_A09G1509* encoding glutathione transferase played a positive role in regulating tobacco seed germination and seedling growth. Furthermore, *Gh_A09G1509* might promote seed germination and seedling establishment through regulating glutathione metabolism in the imbibitional seeds. Our findings provide unprecedented information for deciphering the genetic basis of seed germination and performing molecular breeding to improve field emergence through genomic selection in cotton.

## Introduction

Cotton is an important fiber crop and a valuable oilseed crop. Among the cultivated species, upland cotton (*Gossypium hirsutum* L.) has the largest cultivated areas and plays an essential part in daily life and global textile industry (Fang et al., 2017; Naoumkina et al., 2019). Seed germination is the beginning of the life cycle of seed plants, as well as an important link for high-yield and resistance breeding of cotton. Seed vigor is an important character of seed quality that reflects seed germination rate and uniformity, as well as seedling emergence and growth in the field. Hence, seedling vigor may influence crop yield through both indirect and direct effects (Ellis, 1992; Foolad et al., 2007; Sun et al., 2007). In modern agricultural cultivation practice, rapid and uniform seed germination is the key factor to maximize crop yield potential. Seeds with high vigor germinate quickly and result in uniform and strong seedlings, which ultimately affect plant growth and development (Foolad et al., 2007). With the rapid application of mechanized cultivation and harvest, the quality and vitality of cotton seeds will be particularly important to achieve precision sowing technology.

Seed vigor is a comprehensive embodiment that involves many aspects from seed germination to seedling growth, such as seed germination rate and uniformity, seedling growth rate, and growth rhythm (Ellis, 1992). Seed germination trait is under multiple-genes control and susceptible to environmental factors (Yu et al., 2019a), and is the main component of seed vigor. In previous studies, there have been reports on seed vigor, seed germination, seed field emergence, exogenous hormones, and hormone signaling pathways that regulate seed germination in cotton and other species (Zhang et al., 2014; He et al., 2019; Xiao et al., 2019; Yu et al., 2019a,b; Song et al., 2020; Zhou et al., 2020). In cotton, cold atmospheric-pressure plasma (CAP) treatment improved seed germination and chilling tolerance (Groot et al., 2018). Matrix-localized heat shock protein GhHSP24.7 mediated seed germination *via* temperature-dependent reactive oxygen species (ROS) germination (Ma et al., 2019). In rice, *OsIPMS1* affected seed vigor associated with amino acid and energy metabolism (He et al., 2019), *osa-miR164c* and *osa-miR168a* played a key role in regulating seed vigor in transgenic plants (Zhou et al., 2020). In soybean, quantitative proteomic analyses of two low phytic acid mutants showed that the high germination rate in the TW-1-M might be strongly attributed to ROS-related and plant hormone-related genes (Yu et al., 2019a). In addition, exogenous hormones and hormone signaling pathways have also been reported to play a pivotal role in the regulation of seed germination and dormancy. For instance, exogenous melatonin promoted seed germination and osmotic regulation under arid and salt stress (Xiao et al., 2019; Bai et al., 2020; Chen et al., 2020). *AtPER1* reduced primary seed germination *via* suppressing ABA catabolism and promoting GA biosynthesis in Arabidopsis seeds (Chen et al., 2019). *OsRACK1A* positively regulated seed germination by means of changing the endogenous amounts of ABA and ROS, as well as their interplay (Zhang et al., 2014). *OsMFT2* was involved in the regulation of ABA signaling-mediated seed germination through interacting with *OsbZIP23/66/72* in rice (Song et al., 2020).

Glutathione S-transferases (GSTs) are multifunctional enzymes that play important roles in biological processes such as plant development, metabolism and abiotic, and biotic stress responses by catalyzing the conjugation of electrophilic substrates with glutathione (GSH), thereby reducing their toxicity (Dixon et al., 2002; Moons, 2005; Frova, 2006; Kao et al., 2016). It has been shown that GSTs promote seed germination under abiotic stresses. Seed germination and seedling growth of *GST* overexpressing tobacco was significantly improved under stressful conditions (Roxas et al., 1997). Overexpression of *SbGSTU* in tobacco enhanced seed germination under salt stress (Jha et al., 2010). *Atgstu17* regulated seed germination by the combined effect of GSH and ABA (Chen et al., 2012), and GSH treatment improved germination after seed dehydration (Kalemba and Ratajczak, 2018). The functional deficiency of *AtGSTU7* resulted in increased GSH content and decreased H_2_O_2_ content in germinating seeds, and GSH was involved in seed germination under ABA treatment, implying that *AtGSTU7* involvement in seed germination was mediated by GSH-ROS homeostasis and ABA signaling (Wu et al., 2020).

In the past few years, QTL mapping based on biparental linkage analysis has become an effective approach to identify seed germination related genes in many crops, such as rice (Cui et al., 2002; Jiang et al., 2017; Yang et al., 2019; Jiang et al., 2020), barley (Moursi et al., 2020), and *Brassica rapa* (Basnet et al., 2015), but it was usually limited by the number of markers that could be employed along the chromosomes. As an excellent complement to QTL, GWAS is an effective method to detect mark-trait association (Zhao et al., 2011; Yu et al., 2017), it has been successfully implemented in rice (Sales et al., 2017; Shi et al., 2017; Yang et al., 2019), maize (Hu et al., 2017), oat (Huang et al., 2020), *Brassica napus* (Hatzig et al., 2015; Tan et al., 2017), soybean (Zhou et al., 2015; Liu et al., 2020), and other crops during germination for the identification of single nucleotide polymorphism (SNP) loci and candidate genes for various ecological and agricultural traits. In conclusion, GWAS has been successfully applied to identify the potential candidate genes underlying important agronomic traits with high-density SNPs from diverse germplasms. However, there are few studies on the rapid and accurate identification of a large number of candidate genes for seed germination in cotton, and the seed germination mechanism is still unclear. In addition, previous studies have shown that there is a very significant correlation between seed germination potential and field seedling emergence (Chen, 2012; Xie et al., 2019), and the germination rate measured in sand bed is positively correlated with the seedling emergence rate in the field (Wang, 2007). Therefore, GWAS analysis based on genotypic and phenotypic data for large-scale accessions and SNP markers should provide a powerful strategy to detect candidate genes and unravel the molecular mechanism for seed germination that is important for cotton improvement.

In the present study, we performed a GWAS for seed germination traits based on 3,665,030 SNPs from a core collection consisting of 419 diverse germplasm resources in *G. hirsutum* L. (Ma et al., 2018). The objectives were to identify SNPs significantly associated with germination capacity and candidate genes, providing useful information for better understanding the genetic mechanism of cotton seed germination so as to facilitate molecular breeding with increased field emergence rate and precision sowing.

## Materials and Methods

### Plant Materials

In this study, a core collection comprising of 419 upland cotton accessions was used to conduct GWAS. The 419 accessions had abundant phenotypic variation and were used to conduct GWAS for fiber quality, yield, and phosphorus deficiency tolerance in the previous study of our group (Ma et al., 2018; Gu et al., 2020). Among which, 317 accessions were collected from different provinces of China and the remaining accessions were derived from major cotton-growing countries, including the United States, the former Soviet Union, Pakistan, Turkey, Australia, Mexico, Brazil, Chad, Uganda, Sudan, Bulgaria, and Spain.

### Identification of Seed Germination Relative Traits and Statistical Analysis

After the cotton seeds were delinted by sulfuric acid, 400 full and uniform seeds were chosen from each accession. Four replicates with 100 seeds each were used for each accession. The seeds were placed evenly into a germination chamber containing 800 g dry quartz sand, then covered the seeds with 250 g of dry quartz sand, finally added 250 mL double-distilled H_2_O. The germination chambers were placed in a culture room with 25/20°C temperature and 16/8 h light/dark regime during the period of seed germination. Seeds were considered as germinated when the radicle broke through the seed coat. Seedlings were considered to be established when the root reached half of the seed length. We counted the germinated seeds from the 3rd to the 7th day. Germination potential (GP) refers to the ratio of the number of normal germination seeds to the number of tested seeds in the initial stage of seed germination, usually specified as 3 days, that is, the germination rate (%) of the initial count (Yang et al., 2019; Yuan et al., 2019). GP indicates the speed of germination and the strength of seed vigor. Germination rate (GRA) refers to the proportion of all normal germinated seeds to the number of tested seeds at the end of the germination test, usually specified as 7 days (Yang et al., 2019; Yuan et al., 2019). A high seed germination rate means that there are more viable seeds and more seedlings emergence after sowing. Germination index (GI) represents the sum of the ratio of the number of germinated seeds per day to the corresponding germinating days. GI is calculated based on the formula: GI =Σ(*Gt*/*Dt*). In the formula, Dt refers to the number of days to germinate; Gt is the number of seeds germinated per day corresponding to Dt (Yuan et al., 2019). Vigor index (VI) is a comprehensive reflection of seed germination rate and growth, VI = GI × S, here, S refers to the length (cm) or weight (g) of normal seedlings in a certain period (Yang et al., 2019; Yuan et al., 2019). In this study, S is calculated by mass. Statistical analysis of the GP, GRA, GI, and VI were performed with SPSS 20.0. All of the phenotypic data from 419 cotton accessions were used to calculate the frequency distribution of each trait and descriptive statistics.

### Genome-Wide Association Study and the Identification of Candidate Genes

Association analysis was performed by the genome-wide efficient mixed model association (GEMMA) package using the following equation: *y* = *Xα* + *Sβ* + *Kμ* + *e*. Here, *y* represents the phenotype; α and β are fixed effects, representing marker and non-marker effects, respectively; μ represents unknown random effects; and *X*, *S*, and *K* are the incidence matrices for α, β, and μ, respectively; and *e* is the vector of random residual effects. The top three PCs were used to build up the *S* matrix for population-structure correction. The matrix of simple matching coefficients was used to build up the *K* matrix. The analyses were implemented in the GEMMA software package (Zhou and Stephens, 2012). According to the Bonferroni correction principle, −log_10_ (*P*) > 6.59 (*P* = 1/*n*, *n* is the number of SNPs in this study) is too stringent that we could not find the significant SNPs for four traits with this threshold. Thus, −log_10_ (*P*) > 5.0 was used to identify significant SNP markers with seed germination related traits (Song et al., 2019; Abdelraheem et al., 2021). We identified candidate genes within 300-kb flanking significant-associated SNP loci as putative candidates based on the decay of LD (Ma et al., 2021) and conducted gene annotation based on *G. hirsutum* TM-1 genome (Zhang et al., 2015). LD block identification was performed for associated SNPs using Haploview 4.2 software (Bashir et al., 2018). Gene Ontology (GO) enrichment and Kyoto Encyclopedia of Genes and Genomes (KEGG) pathway analysis were carried out for all candidate genes (Xie et al., 2011).

### Expression Profile of Candidate Genes

To screen the possible candidate genes involving in the seed germination, the expression level of these genes were analyzed based on the transcriptomic data from the seeds soaking in water for 0, 5, and 10 h. Transcriptome data of gene expression in different tissues and different germination periods was obtained from a previous study (Zhang et al., 2015) and the fragments per kilobase of transcript per million mapped reads (FPKM) values of putative candidate genes were extracted for comparison between different tissues and different germination periods. The heat maps were generated with HemI version 1.0.^1^

In order to verify the expression trend of the candidate genes, two cotton varieties with fast germination (Jinmian2) and slow germination (Qunkemian) were chosen for expression analysis with qRT-PCR. The seeds of selected varieties were spread evenly on the sterilized gauze in the germination chamber, sprayed enough sterile water to keep seeds moist during the whole germination period. Imbibitional seeds were taken at 0, 6, 12, 24, 36, 48, 60, and 72 h, respectively. The samples were immediately frozen in liquid nitrogen and stored at −80°C for RNA extraction with three replicates. Total RNA was extracted with an EASYspin Plus Plant RNA purification kit (Aidlab, Beijing, China). Total cDNA was synthesized with the PrimerScriptTM RT Reagent Kit together with gDNA Eraser (TaKaRa, Dalian, China). Quantitative real-time PCR was performed with Auge Green™ qPCR Master Mix (US EVERBRIGHT RINC) on an ABI 7500 Real-Time PCR machine. The qRT-PCR mixtures consisted of 10 μl of AugeGreen™ Master Mix (US EVERBRIGHT RINC), 2.0 μL of ROX reference dye, 2.0 μL of cDNA, 1.0 μL of primers, and ddH_2_O supplemented the volume to 20 μL. The reactions were amplified at 95°C for 30 s, followed by 40 cycles of 95°C for 5 s, 55°C for 30 s, and 72°C for 30 s. All the reactions were performed as three technical replicates. Relative gene expression levels were calculated with the 2^–ΔΔCT^ method. The primers used are listed in Supplementary Table 1.

### Gene Cloning and Plant Transformation

The open reading frame (ORF) of *Gh_A11G0176* was obtained through PCR using cDNAs synthesized from RNA, the amplified products were subsequently cloned into the pGreen vector which was driven by the cauliflower mosaic virus (CaMV) 35S promoter. Using the floral dip method (Clough and Bent, 1998), the recombinant vector was transformed into *Arabidopsis thaliana* Columbia type by *Agrobacterium tumefaciens* GV3101. Transgenic plants were obtained by screening successive generations on Basta. PCR was utilized to identify homozygous T_3_ transgenic lines, which were subsequently employed in further investigations. For the seed germination assay, thirty plump seeds were surface-sterilized for 5 min in 30% NaClO and 1 min in 75% ethanol, washed at least five times with sterile water, plated on Murashige and Skoog (MS) solid medium (with 1% sucrose) and stratified at 4°C for 48 h, then grown at 22°C under long-day conditions (16/8 h light/dark). For each germination assay, biological triplicates were performed, and germination ability was observed every 12 h until all seeds germinated after 24 h.

In addition, another target gene *Gh_A09G1509* was selected to validate with overexpressing transgenic tobacco which was previously acquired by our research group (Li et al., 2019). Approximately 50 plump seeds each from the wild-type (WT) and over-expression (OE) tobacco were surfaced sterilized and plated on MS media (pH 5.7–5.9) at 28°C in the incubator under long-day conditions (16/8 h light/dark), with independent biological triplicates. Germination ability was observed every 24 h until all seeds germinated after 2 days, and the method was the same as above.

### Measurement for Hormone Content

Gibberellin (GA) and abscisic acid (ABA) were extracted from 0.1 g germinating seeds using 1 mL precooling reagent one (methanol: water: acetic acid = 80: 20: 1) overnight at 4°C and centrifuged at 8,000 g at 4°C for 10 min. The residue was collected and added 0.5 mL reagent one (methanol: water: acetic acid = 80: 20: 1) for 2 h, then the supernatant was collected after centrifugation. Two supernatants were combined for drying with nitrogen at 40°C until no organic phase remained. The supernatants were added 0.5 mL reagent two (petroleum ether) to extract and decolorize three times at 60–90°C, the upper ether phase was discarded, and the lower aqueous was adjusted to pH 2.8 with reagent three (saturated citric acid aqueous solution). Next, the mixture was extracted three times with equal volume of reagent four (ethyl acetate). After the organic phase with nitrogen was blown dry, the extraction was diluted to 0.5 mL with reagent five (methanol) through vortex oscillation, and filtered by 0.22-μm membrane filter. A high-performance liquid chromatography (HPLC) device was used to analyze the final filtrate solution. The content of GA and ABA were determined at 210 and 254 nm, respectively, and was expressed as μg/g fresh weight (FW).

### H_2_O_2_ Extraction and Analysis

The H_2_O_2_ level was determined using commercial assay kits according to the manufacturer’s instructions (Suzhou Keming Bioengineering Company, China). Approximately 0.1 g FW of each sample was quickly put into precooled acetone (4°C) and homogenized on ice bath. The reaction solutions were then mixed into the homogenate. The mixture was centrifuged at 8,000 g at 4°C for 10 min, and the absorbance of the supernatant was measured at 415 nm right away. The H_2_O_2_ content was expressed as μmol/g FW.

### Protein, Glucose, and Amylase Activity Assays

Protein, glucose, and amylase activity were measured using commercial assay kits following the manufacturer’s instructions (Suzhou Keming Bioengineering Company, China). The levels of protein and glucose were expressed as mg/g FW. One unit (U) of amylase is defined as 1 mg of reducing sugar produced by enzyme in 1 g FW of the sample in 1 min at 40°C. The activity of α-amylase were expressed as U/g FW.

### Extraction and Measurement of Glutathione and Enzymes Related to Glutathione Metabolism

Glutathione, oxidized glutathione (CSSG), glutathione peroxidase (GPX), glutathione reductase (GRE), and GST were measured using commercial assay kits following the manufacturer’s instructions (Suzhou Keming Bioengineering Company, China). The levels of GSH and CSSG were expressed as μ mol/g FW and nmol/g FW, respectively.

Glutathione reductase activity was determined following the rate of NADPH oxidation at 340 nm. The GPX activity was calculated by measuring the rate of disappearance of NADPH at 340 nm. One unit (U) of GRE activity and GPX activity was defined as each gram of sample catalyzed the oxidation of 1 nmol NADPH per minute. GST activity was calculated by measuring the increase in absorbance at 340 nm. One unit (U) of GST activity was defined as each gram of sample catalyzed the combination of 1 nmol/L CDNB and GSH per minute. The activities of GRE activity, GPX activity, and GST activity were expressed as U/g FW.

## Results

### Phenotypic Variation of Seed Germination Related Traits

**FIGURE 1:**
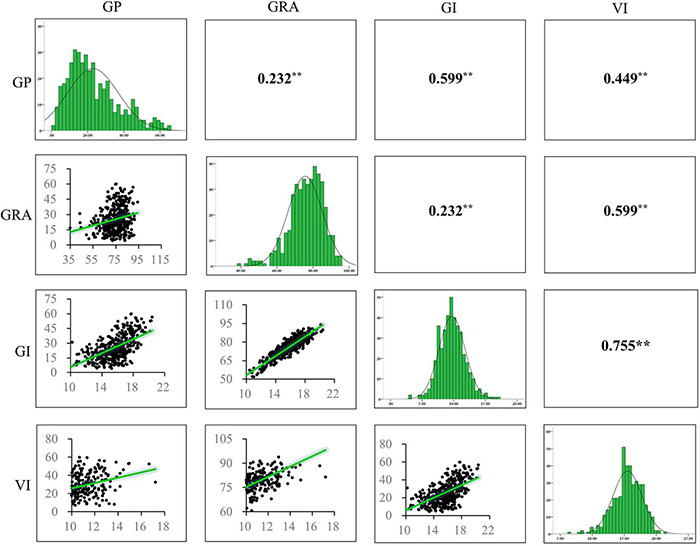
Frequency distribution of phenotypic variation of four seed germination related traits and correlation coefficients among the traits in 419 accessions. ** indicates significance at the *P* < 0.01 level (two-tailed *t*-test). GP, germination potential; GRA, germination rate; GI, germination index; VI, vigor index.

We analyzed the phenotypic variation of seed germination relevant traits including GP, GRA, GI, and VI. The results showed that all the traits displayed broad variations. The GP ranged from 4.00 to 60.00% with an average of 25.41%, the GRA ranged from 35.00 to 94.00% with an average of 75.04%, the GI ranged from 7.52 to 20.41 with an average of 15.55, and the VI ranged from 3.44 to 17.21 with an average of 9.85. The coefficient of variation (CV) of GP, GRA, GI, and VI were 47.82, 11.75, 13.21, and 18.98%, respectively (Table 1). High correlations were observed among these seed germination traits (Figure 1). GP was significantly (*P* < 0.001) and positively correlated with GRA (*r* = 0.232^**^), GI (*r* = 0.599^**^) and VI (*r* = 0.449^**^), and GRA was significantly positively correlated with GI (*r* = 0.910^**^) and VI (*r* = 0.677^**^), which may facilitate the identification of pleiotropic gene in response to seed germination. Moreover, the phenotypic distribution of these traits displayed continuous variation (Figure 1), indicating that seed germination related traits were quantitatively inherited.

**TABLE 1:** Phenotypic variation statistics for seed germination traits.

Variable	Minimum	Maximum	Mean	Std. deviation	CV%	Skewness	Kurtosis
GP, germination potential (%)	4.00	60.00	25.41	12.15	47.82	0.597	−0.205
GRA, germination rate (%)	35.00	94.00	75.04	8.82	11.75	−0.865	1.372
GI, germination index	7.52	20.41	15.55	2.05	13.18	−0.527	0.467
VI, vigor index	3.44	17.21	9.85	1.87	18.98	0.247	0.664

### Identification of Significantly Associated Single Nucleotide Polymorphisms and Candidate Genes Related to Seed Germination Traits

**FIGURE 2:**
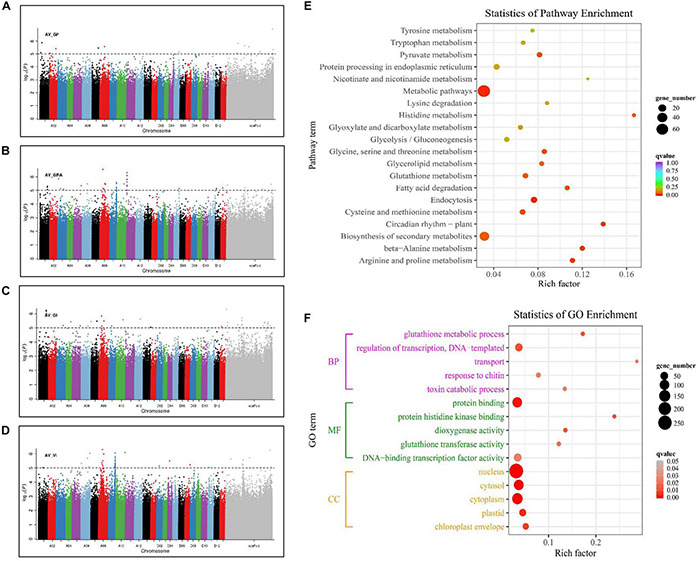
Manhattan plots of the four seed germination-related traits and GO function and KEGG pathway enrichment of candidate genes. **(A)** GP. **(B)** GRA. **(C)** GI. **(D)** VI. **(E)** Statistics of KEGG pathway enrichment analysis of 723 genes detected in GWAS. **(F)** Gene Ontology (GO) functional enrichment analysis of 723 genes detected in GWAS. Each point in the figure represents a GO channel, and the channel name is shown on the left axis. The abscissa is the enrichment factor, which represents the ratio of the proportion of proteins annotated to this pathway in the differentially expressed protein to the proportion of proteins that are annotated to a pathway of the species protein. The larger the enrichment factor, the more reliable the significance of the enrichment of differential proteins in this pathway.

Genome-wide association study results showed that 92 significantly associated SNPs were identified, of which, 10 and 35, 24 and 36 were associated with GP, GRA, GI, and VI, respectively, and distributed on 20 chromosomes (Figures 2A–D). There were 69, 10, and 13 SNPs in the A-subgenome, D-subgenome, and scaffolds, respectively (Supplementary Table 2). Among them, the maximum number of associated SNPs was detected on chromosome A09 (20), and no significant SNPs were detected on chromosome D01, D03, D06, D08, D09, or D10. In addition, 13 associated SNPs located on chromosomes A01, A05, A08, A09, A10, and D13 were observed in multiple traits (Supplementary Table 3). Among these, four SNPs on chromosome A01 were found to be significantly associated with GI and GRA, two SNPs on chromosome A08 with GI and GRA, four SNPs on chromosome A09 with GRA and VI, and three SNPs on chromosome A05, A10, and D13 with GRA and GI.

To explore the potential candidate genes related to seed germination, we selected a 300-kb region flanking significant SNPs according to the linkage decay value (Ma et al., 2018). A total of 723 candidate genes were identified, of which, 569 and 154 were located on the A-subgenome and D-subgenome, respectively (Supplementary Table 4). Their expression were analyzed on the basis of the transcriptome data from cottonFGD (Supplementary Figures 1, 2). For the A-subgenome, the expressions patterns of all the genes could be divided into five types according to the expression specificity from different tissues and different germination periods. Type I were mainly expressed in seed, and their expression gradually decreased with the extension of seed imbibition time, such as *Gh_A11G0176, Gh_A11G0179, Gh_A11G0184*, and *Gh_A09G1510*. Type II displayed higher expression in root than in seed. Type III was specifically expressed in cotyledon. Type IV was less expressed in seed, but their expressions gradually increased with the extension of seed imbibition time, such as *Gh_A09G1508* and *Gh_A09G1509*. Type V was mainly expressed in cotyledon and seed, and the expression gradually decreased with the extension of seed imbibition time, such as *Gh_A11G0177* (Supplementary Figure 1). Similarly, the gene expressions in the D-subgenome were also divided into five types the same as to those in the A-subgenome (Supplementary Figure 2). KEGG pathway analysis was performed to display the top 20 significantly enriched pathway, and thereinto, glutathione metabolism (KO00480) and circadian rhythm (KO04712) are related to the multifunction of GST and seed germination (Figure 2E). GO enrichment analysis was conducted to further infer the functions of candidate genes (Figure 2F). At *P* < 0.05 and gene number > 3,723 candidate genes were classified into three major categories: biological process (BP), molecular function (MF), and cell component (CC). In the biological process, transport (GO: 0006810), glutathione metabolic process (GO: 0006749), and toxin catabolic process (GO: 0009407) were the most functional terms associated with seed germination. In the molecular function, protein binding (GO: 0005515), dioxygenase activity (GO: 0051213), and glutathione transferase activity (GO: 0004364) were the significantly enriched items. In the cellular component (CC) category, chloroplast envelope (GO: 0009941), and cytoplasm (GO: 0005737) were the two most prevalent functional terms. GRE was mainly distributed in chloroplast but also in cytoplasm, which related to the stress tolerance and seed germination (Ding et al., 2009; Gill and Tuteja, 2011).

### Functional Analysis of Candidate Genes

**FIGURE 3:**
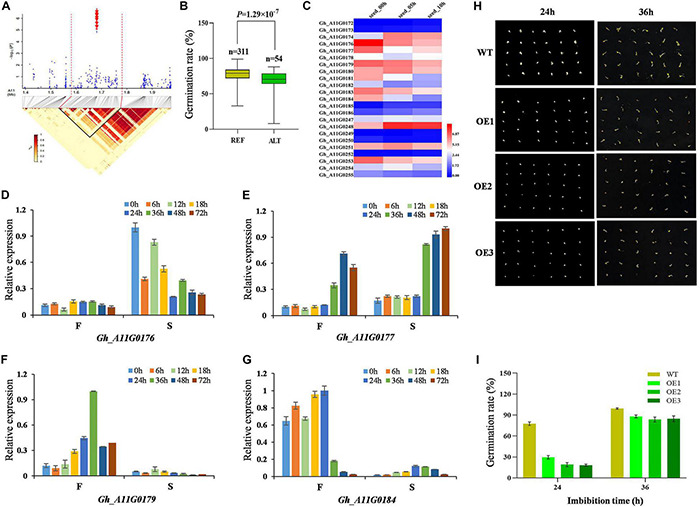
Identification and expression analysis of candidate genes related to seed germination on chromosome A11. **(A)** Local Manhattan plot (top) for GRA and LD heat map (bottom) surrounding the peak region (1.4–2.0 Mb) on A11. Red dotted lines indicate the candidate region, and the red arrows indicate A11:1681226, A11:1681264, A11:1681275, A11:1681285, A11:1681287, A11:1681352, A11:1681419, A11:1681428, and A11:1681444. **(B)** Box plots for GRA, based on the significantly associated SNP on A11, n indicates the number of accessions with the same genotype. REF, the reference TT; ALT, the alternate TC. Significant difference was analyzed with a two-tailed *t*-test. **(C)** Heat map of the candidate gene expression of chromosome A11 at different cotton seed imbibition stage based on the FPKM value. Red indicates high expression, and blue indicates low expression. **(D–G)** Relative expression of four candidate genes (*Gh_A11G0176*, *Gh_A11G0177*, *Gh_A11G0179*, and *Gh_A11G0184*) between F (cotton variety with fast germination: Jinmian2) and S (cotton variety with slow germination: Qunkemian) via qRT-PCR at 0, 6, 12, 24, 36, 48, 60, and 72 h, *Ghhistone3b* was used as an internal control. The data were normalized to the maximum value. The error bars indicate ± SE; *n* = 3 independent biological replicates. **(H)** Germination phenotype of *Gh_A11G0176* overexpressing lines and the WT at the seed germinating stage 24 and 36 h in Arabidopsis. WT, wild-type plants. **(I)** GRA of overexpressing lines and the WT in Arabidopsis.

On chromosome A11, we focused on the locus mapped from 1.4 to 2.0 Mb, where a locus (A11:1681419) was significantly associated with GRA (Figure 3A), and the two genotypes of the locus showed significant difference (Figure 3B). A total of 135 candidate genes were identified within the LD region. We focused on the expression of 23 candidate genes in the 50-kb region flanking the significant SNP (Figure 3C). These genes were involved in heat shock protein 70 (Hsp 70) family protein, translation elongation factor EFG/EF2 protein, thioredoxin family protein, AT motif nuclear localization protein, and other proteins that performed molecular functions. It can be seen from the heat map that the expression levels of *Gh_A11G0176*, *Gh_A11G0177*, *Gh_A11G0179*, and *Gh_A11G0184* gradually decreased with the extension of seed imbibition time (Figure 3C). Their expression patterns were analyzed *via* qRT-PCR using a fast germinating and a slow germinating variety at 0, 6, 12, 18, 24, 36, 48, and 72 h after imbibition (HAI). Compared with the fast germinating variety, *Gh_A11G0176* and *Gh_A11G0177* displayed a higher expression in the slow germinating variety (Figures 3D,E). However, *Gh_A11G0179* and *Gh_A11G0184* exhibited higher expression in the fast germinating varieties (Figures 3F,G). To understand the function of target gene *Gh_A11G0176* encoding Hsp70-Hsp90 organizing protein 3, we overexpressed the gene in Arabidopsis and obtained the homozygous lines. The OE Arabidopsis lines and WT were put on MS medium for germination assays. The results showed that the germination rates of OE were markedly reduced compared with WT during germination (Figures 3H,I), that is, the OE of *Gh_A11G0176* caused delayed germination. At 24 HAI, the germination rate of WT (78%) was approximately 4.3 times that of OE-3 (18%). At 36 HAI, most of the OE and WT have already germinated, and the germination rates of WT and OE were almost the same.

**FIGURE 4:**
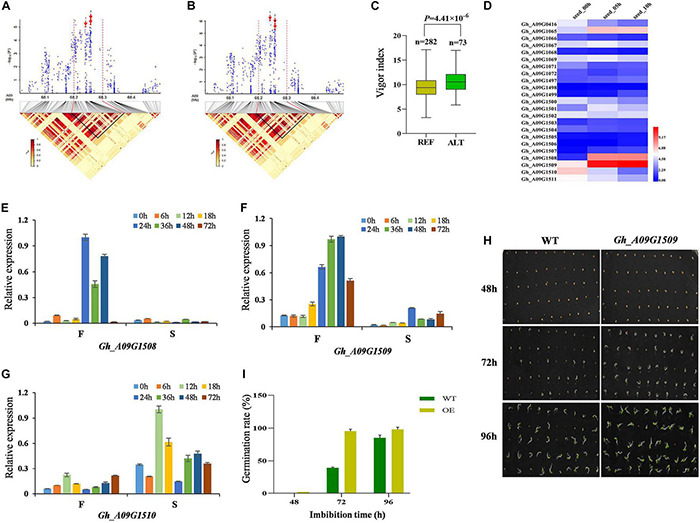
Identification and expression analysis of candidate genes related to seed germination on chromosome A09. **(A)** Local Manhattan plot (top) for GRA and LD heat map (bottom) surrounding the peak region (68.0–68.5 Mb) on A09, and red arrow 1, 2, and 3 indicate the position of associated SNP A09:68240653, A09:68257990, and A09:68258389, respectively. **(B)** Local Manhattan plot (top) for VI and LD heat map (bottom) surrounding the peak region (68.0–68.5 Mb) on A09. **(C)** Box plots for VI, based on the significantly associated SNP on A09, *n* indicates the number of accessions with the same genotype. REF, the reference GG; ALT, the alternate GA. Significant difference was analyzed with a two-tailed *t*-test. **(D)** Heat map of the candidate gene expression of chromosome A09 at different cotton seed imbibition stage based on the FPKM value. Red indicates high expression, and blue indicates low expression. **(E–G)** Expression analysis of three candidate genes (*Gh_A09G1508*, *Gh_A09G1509*, and *Gh_A09G1510*) between F (cotton variety with fast germination: Jinmian2) and S (cotton variety with slow germination: Qunkemian) via qRT-PCR at 0, 6, 12, 24, 36, 48, 60, and 72 h, *Ghhistone3b* was used as an internal control. The data were normalized to the maximum value. The error bars indicate ± SE; *n* = 3 independent biological replicates. **(H)** Germination phenotype of *Gh_A09G1509* overexpressing lines and the WT at the seed germinating stage 48, 72, and 96 h in tobacco. WT, wild-type plants. **(I)** GRA of overexpressing lines and the WT in tobacoo.

On chromosome A09, we focused on the locus mapped from 68.0 to 68.5 Mb with significant signals, of which SNP A09:68240653 was significantly associated with GRA and VI (Figures 4A,B). Ninety-five candidate genes were identified (Supplementary Table 4). The accessions carrying the alternate genotype germinated faster than those with the reference (Figure 4C). We analyzed the expression of 23 candidate genes within the 50-kb region flanking the significant SNP and found that glutathione transferase encoding genes, *Gh_A09G1508* and *Gh_A09G1509*, displayed an increased trend with the extension of seed imbibition time excluding *Gh_A09G1510* (Figure 4D). We further analyzed the expression of three genes using a fast germinating and a slow germinating variety at 0, 6, 12, 18, 24, 36, 48 and 72 HAI by qRT-PCR. The results showed that the expression of *Gh_A09G1508* and *Gh_A09G1509* was higher in the fast germinating variety, however, *Gh_A09G1510* was higher in the slow germinating variety (Figures 4E–G). Moreover, the OE of *Gh_A09G1509* in tobacco resulted in faster seed germination and seedling growth as well as longer hypocotyls at 3 days after imbibition (DAI) compared with WT. The cotyledons of transgenic tobacco unfolded earlier than WT at 4 DAI (Figures 4H,I), indicating that *Gh_A09G1509* promoted the plant seed germination.

### Effects of Gh_A09G1509 on Endogenous Phytohormones, H_2_O_2_, Starch Mobilization, and Soluble Sugar Content During Germination

**FIGURE 5:**
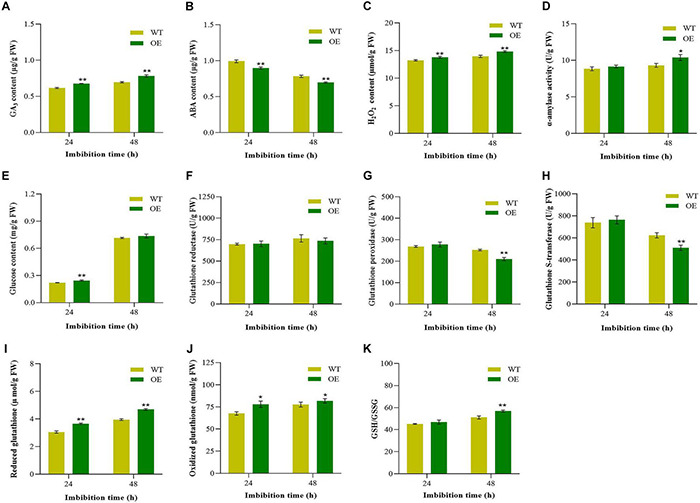
Comparison of endogenous hormones, H_2_O_2_, starch mobilization, glucose, and glutathione metabolism between the overexpressing lines and the WT during seed germination of tobacco. **(A)** GA_3_ content; **(B)** ABA content; **(C)** H_2_O_2_ content; **(D)** α-amylase activity; **(E)** Glucose content; **(F)** Glutathione reductase; **(G)** Glutathione peroxidase; **(H)** Glutathione S-transferase; **(I)** Reduced glutathione; **(J)** Oxidized glutathione; **(K)** the ratio of reduced glutathione and oxidized glutathione. Each column represents the means ± SD. * and ** indicate the significant difference compared to WT at 5 and 1% levels, respectively.

Abscisic acid and gibberellin are the key endogenous substances that work antagonistically in the regulation of seed germination. Furthermore, H_2_O_2_ in seeds as a signal can promote germination and seedling growth (Barba-Espin et al., 2010; Katsuya-Gaviria et al., 2020), which was induced by GA but suppressed by ABA (Ishibashi et al., 2012). Therefore, endogenous GA_3_, ABA, and H_2_O_2_ contents were measured during seed germination. Compared with WT, the OE transgenic tobacco showed significantly higher GA_3_ contents (Figure 5A). For ABA contents, it was significantly lower in the transgenic tobacco at 48 HAI (Figure 5B). At 48 HAI, the hypocotyl of the tobacco broken through the seed coat and reached to protrusion. As similar to GA_3_, the endogenous H_2_O_2_ was significantly higher at 48 HAI in transgenic tobacco than WT (Figure 5C). Imbibition and starch hydrolysis are the critical steps during seed germination. We further compared the changes of α-amylase and glucose contents between the transgenic tobacco and the WT. The content of α-amylase was significantly higher in the OE lines at 48 HAI (Figure 5D). Furthermore, we observed that the content of glucose appeared an apparent increase at 48 HAI (Figure 5E). These results indicated that *Gh_A09G1509* regulated seed vigor through adjusting the relative contents of endogenous phytohormones and altering starch hydrolysis and glucose contents in germinating seeds.

### Changes of Substances Related to Glutathione Metabolism During Germination

Doubled glutathione improved the germination capacity (Kalemba and Ratajczak, 2018), and GRE and GPX regulated the balance between the reduced and oxidized forms of glutathione in plants (Zhao et al., 2021), which is crucial for cellular redox state homeostasis and plant development (Hernandez et al., 2015). Since *Gh_A09G1509* belonged to *GST* gene family, the related substances of glutathione metabolism were measured during germination. There was no significant difference in GRE between the OE tobacco plants and the WT (Figure 5F), but the activities of GPX and GST decreased during germination, and the activities of GPX and GST in the OE plants were significantly lower than those in WT plants at 48 HAI (Figures 5G,H), when the hypocotyl broken through the seed coat. Compared with WT, the content of GSH and GSSG were also significantly higher in transgenic tobacco at 48 HAI (Figures 5I,J). Similarly, the ratio of GSH/GSSG in the OE tobacco plants was significantly higher during the whole germination period, especially at 48 HAI (Figure 5K). The results suggested that *Gh_A09G1509* might facilitate glutathione metabolism during seed germination.

## Discussion

Rapid and uniform seed germination under various conditions is an agronomically important trait for high yield of crops (Foolad et al., 2007). Seeds with high vigor have obvious production advantages and potential, thus, it is critical to identify and utilize seed vigor related genes for improving seed vigor (He et al., 2019). The development of seedlings with enhanced vigor could allow for earlier planting, extended agricultural growing seasons for crops and the expansion of crop production in marginal locations (Roxas et al., 1997). In the present study, a total of 92 significantly associated SNPs located within or near 723 genes were identified for seed germination traits on the basis of a core collection containing 419 cotton accessions with 3.66 million high-quality SNPs. More associated SNPs were located in the A-subgenome than D-subgenome. The candidate genes were involved in various metabolic pathways, including toxin metabolism, toxin decomposition, glutathione metabolism, and circadian rhythms with plants. Some of these candidate genes related to germination have been identified in rice and other plant species (Guo et al., 2011; Kaur et al., 2015; Wang et al., 2015). The results provide new insights into the genetic basis of seed germination vigor and molecular tools for crop improvement in cotton.

On chromosome A09 and A11, *Gh_A09G1499*, *Gh_A09G1610*, *Gh_A11G0179*, and *Gh_A11G0252* belonged to the protein kinase superfamily which played fundamental roles in the modulation of plant growth and development, including seed development, dormancy and germination, seedling and root growth, flowering, fruit development, and ripening and leaf senescence (Halford and Hey, 2009; Wang et al., 2020). Overexpression of the receptor protein kinase gene *ZmRLK*7 in Arabidopsis reduced the plant height, organ size (e.g., petals, silique, and seeds) and seed weight (He et al., 2020). The kinase-associated protein phosphatase (KAPP) in Arabidopsis was negatively involved in ABA-mediated seed germination and early seedling growth (Lu et al., 2020). *SnRK*2 regulated key traits of crop improvement and production such as seed maturation and germination *via* ABA-dependent or ABA-independent pathways (Mao et al., 2020).

*Gh_A11G0180* and *Gh_A09G1503* encoded thioredoxin (Trx) family proteins. During seed germination, Trx acts as a signal in early germination to promote amylase and proteinase activities and initiated the hydrolysis of storage materials, thereby promoting seed germination (Smiri et al., 2010; Guo et al., 2011). Trx has been observed to accelerate germination and α-amylase synthesis in some transgenic studies (Cho et al., 1999; Wong et al., 2002; Kim et al., 2003). Trx *h* present in starchy endosperm functions in germination and early seedling development in cereals (Jiao et al., 1992; Kobrehel et al., 1992; Besse et al., 1996; Bewley, 1997; Cho et al., 1999; Wong et al., 2003). Overexpression of *Trx* in barley (*Hordeum vulgare*) endosperm accelerated germination (Wong et al., 2002), and suppressed expression of *Trx* in wheat (*Triticum aestivum*) inhibited germination (Guo et al., 2007).

*Gh_A09G1506* encoding a seed storage protein (SSP) was deposited in the protein bodies of developing seeds and subsequently utilized during the germination of plant as a source of nitrogen and carbon (Kawakatsu et al., 2010). In peanut, embryonic properties could be suppressed *via* repression of *SSP* genes during germination (Yang et al., 2015). Ectopic expression analysis of *PtCP5* showed decreased storage protein accumulation, delayed seed germination, and seedling development in *OX-PtCP5* transgenic Arabidopsis (Liu et al., 2021). Research on rice mutants and transgenic complementary mutants suggested that *OsTudor-SN* functioned in post-transcriptional regulation of storage protein expression and seed development (Chou et al., 2019).

Four candidate genes, *Gh_A11G0171*, *Gh_A11G0174*, *Gh_A11G0176*, and *Gh_A11G0181*, belonged to the heat shock protein (HSP) family that responded to abiotic stress and protected plants from adverse environmental effects (Charng et al., 2006; Sun et al., 2012). Recent studies showed that HSP played an important role in seed germination. Overexpression of *ZmHSP16.9* in transgenic tobacco increased seed germination rate (Sun et al., 2012), and overexpression of *CaHsp*25.9 in *A. thaliana* resulted in increased germination and root length under abiotic stress (Feng et al., 2019). *CsHSP* transformed plants improved seed germination vigor under heat stress (Wang et al., 2017), and *GhHSP24.7* mediated seed germination *via* thermal sensing. Under rugged environmental conditions, the OsHSP18.2 positively controlled the germination and cotyledon emergence (Kaur et al., 2015). In the present study, we validated the function of *Gh_A11G0176*, and the OE Arabidopsis lines showed delayed germination compared with the WT, indicating *Gh_A11G0176* might play a negative role duringseed germination.

Several GST genes, including *Gh_A09G1508*, *Gh_A09G1509*, *Gh_A09G1510*, *Gh_A11G0199*, and *Gh_A11G0200* were identified in the present study. GSTs are versatile enzymes and catalyze the conjugation of electrophilic substrates to GSH and thus reduce their toxicity (Frova, 2006). In addition to glutathione transferase activity, some GSTs were found to possess glutathione peroxidase activity (Bartling et al., 1993; Xu et al., 2016) or be involved in light-dependent pathways and circadian rhythm changes (Galle et al., 2018). GSTs were activated by a variety of environmental stimuli and were found to perform a direct function in lowering oxidative damage and hazardous compounds produced during xenobiotic metabolism (Dixon et al., 2002; Moons, 2005; Frova, 2006). Seed germination and seedling growth were significantly improved in transgenic tobacco lines that overexpressed plant GST/GPX under stressful conditions (Roxas et al., 1997), while overexpression of *Gh_A09G1509* in tobacco resulted in enhanced Verticillium wilt resistance (Li et al., 2019). In the present study, *Gh_A09G1509* was observed to have a higher expression level in fast germinating varieties than in slow germinating varieties, which was consistent with the transcriptome results. Moreover, *Gh_A09G1509* OE tobacco resulted in faster seed germination and seedling growth as well as longer hypocotyls, indicating that *Gh_A09G1509* played a role in the positive regulation of promoting seed germination.

Seed germination is a complex trait that not only affected by temperature and environment, but also by endogenous hormones, such as ABA and GA. The induction and maintenance of dormancy are favorably regulated by ABA, while germination is enhanced by GA. The time of germination depends on the balance and the physiological interaction between ABA and GA (Savage and Metzger, 2006). Researches in rice showed that *OsIPMS*1 could promote the synthesis of GA_3_ biosynthesis-related amino acids in germinated seeds, which resulted in an increase in the amount of soluble sugars available for glycolysis during seed germination. And then, the tricarboxylic acid cycle (TCA) will be boosted, resulting in increased glycolysis and TCA cycle metabolites, which contributed to quick seed germination and strong seedling growth (He et al., 2019). *OsMFT*2 positively regulated ABA response genes through interacting with *OsbZIP*23/66/72 and negatively regulated seed germination in rice (Song et al., 2020). In cotton, appropriate melatonin may promote seed germination by regulating the endogenous phytohormones GA_3_ and ABA (Xiao et al., 2019). In the present study, it was demonstrated that *Gh_A09G1509* improved seed germination by increasing GA_3_ content and decreasing ABA content, in consistent with the literature described on these hormones (Kucera et al., 2005; Tuan et al., 2018). ROS triggered protein carbonization to release dormancy, more and more evidence subsequently showed that ROS homeostasis was essential for germination (Oracz et al., 2007). Studies barley seeds found that dormant seeds had low ROS content but high ABA content. Seed dormancy and germination may be influenced by changes in the equilibrium between ABA and ROS (Ishibashi et al., 2017). The activated C kinase 1 (RACK1) receptor OsRACK1A positively regulated seed germination by regulating endogenous levels of ABA and ROS, as well as their interplay (Zhang et al., 2014). Additionally, the H_2_O_2_ accumulation might change the hormone balance by increasing GA_*s*_ and decreasing ABA and ethylene, which was crucial for seed dormancy and germination (Barba-Espin et al., 2010, 2011; Jeevan et al., 2015). In the present study, we observed that the H_2_O_2_ levels in overexpressed plants were significantly higher compared to WT during the whole seed germination stage. At the later stage of germination (48 h), GA_3_ induced an increase in the activity of α-amylase which catalyzed the hydrolysis of starch into glucose. Soluble sugars such as glucose, serve as the primary energy source for seed germination (Ding et al., 2012). We speculated that *Gh_A09G1509* may regulate seed germination through GA and reactive oxygen signaling pathways. The interaction between GA and H_2_O_2_ promoted the hydrolysis of starch, which increased the glucose content and thus promoted seed germination.

Glutathione is not only an essential metabolite of plant life, but also plays an important role in protein biosynthesis of plant cells, the ratio of GSH to CSSG is the important indicator reflecting the activity of glutathione in plants (Cairns et al., 2006). At the early stage of seed germination, a high ratio of GSH to CSSG is necessary for the synthesis of proteins required for growth and development (Fahey et al., 1980). During the germination process, GPX can catalyze GSH to GSSG and reduce toxic peroxides to non-toxic hydroxyl compounds, and GST can catalyze the combination of GSH and toxic substances or peroxides to inactivate them, thereby protecting cells from oxidative damage (Roxas et al., 2000; Kalemba and Ratajczak, 2018), and promoting seed germination (Wu et al., 2020). In silver maple, GSH treatment caused less dehydration and increased germination (Kalemba and Ratajczak, 2018). In the present study, the activity of GRE gradually increased, while the activities of GPX and GST gradually decreased with the germinating of tobacco seeds. Moreover, overexpression of GRE in transgenic plants leads to elevated levels of GSH. Thereby, we speculated that *Gh_A09G1509* might promote seed germination and seedling establishment by glutathione metabolism.

## Conclusion

In this study, a total of 92 significantly associated SNPs and 294 expressed genes at seed germination stage were screened out. *Gh_A11G0176* might play a negative role while *Gh_A09G1509* play a positive role in regulating the germination of cotton seeds. *Gh_A09G1509* might regulate seed vigor and seedling establishment mainly *via* glutathione metabolism and H_2_O_2_ level in germinating seeds. This provides a valuable reference for understanding the molecular mechanism and facilitating crop improvement of seed germination in cotton.

## Data Availability Statement

The original contributions presented in the study are included in the article/Supplementary Material, further inquiries can be directed to the corresponding authors.

## Author Contributions

ZM conceptualized and designed the research. AS, ZL, BC, QG, YZ, LW, and GZ performed the experiments. AS, ZS, and BC analyzed the data. AS wrote the manuscript. ZM and XW revised the manuscript. All authors contributed to the article and approved the submitted version.

## Conflict of Interest

The authors declare that the research was conducted in the absence of any commercial or financial relationships that could be construed as a potential conflict of interest.

## Publisher’s Note

All claims expressed in this article are solely those of the authors and do not necessarily represent those of their affiliated organizations, or those of the publisher, the editors and the reviewers. Any product that may be evaluated in this article, or claim that may be made by its manufacturer, is not guaranteed or endorsed by the publisher.

## Footnotes

1
http://hemi.biocuckoo.org/

## Supplementary Material

Supplementary Figure 1 Heat map of the expression of candidate genes in different tissues, including cotyledon, root, and seed at different time periods on the A-subgenome.

Supplementary Figure 2 Heat map of the expression of candidate genes in different tissues, including cotyledon, root, and seed at different time periods on the D-subgenome.
