# Supplementary figures and images for: A Genome Wide Association Study Revealed Key Single Nucleotide Polymorphisms/Genes Associated With Seed Germination in Gossypium hirsutum L
Source: Front Plant Sci. 2022 Mar 16;13:844946. doi: 10.3389/fpls.2022.844946 (PMC8967292; doi:10.3389/fpls.2022.844946)

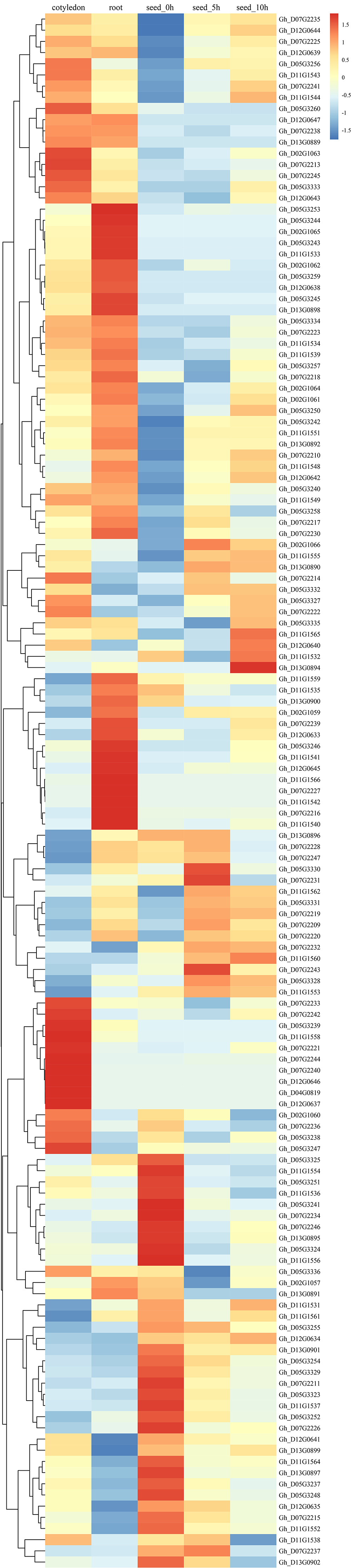

Supplement: Supplementary Figure 2 — Heat map of the expression of candidate genes in different tissues, including cotyledon, root, and seed at different time periods on the D-subgenome. [file Image_2.JPEG]
