# Supplementary material for: A Genome Wide Association Study Revealed Key Single Nucleotide Polymorphisms/Genes Associated With Seed Germination in Gossypium hirsutum L
Source: Front Plant Sci. 2022 Mar 16;13:844946. doi: 10.3389/fpls.2022.844946 (PMC8967292; doi:10.3389/fpls.2022.844946)
Supplement: Supplementary file 3 [file Table_1.docx]

**Table S1.** A list of primers for qRT-PCR.

| Gene ID | Primer name | sequence |
| --- | --- | --- |
| Gh_A09G1508 | Gh_A09G1508-qF | GCCTGCGGTTAGGAAAGCTA |
|  | Gh_A09G1508-qR | GATCGCATCCCCACCAAAGA |
| Gh_A09G1509 | Gh_A09G1509-qF | GCTACCGCTCGATTCTGGAT |
|  | Gh_A09G1509-qR | GCAAGCTTCGTTCGTCACTT |
| Gh_A09G1510 | Gh_A09G1510-qF | TTGCGGTTGAAAGGTGTTCC |
|  | Gh_A09G1510-qR | TGTGGCAAGATGGGATAGGT |
| Gh_A11G0176 | Gh_A11G0176-qF | AAGGAGGAGGTGAAGAAGCC |
|  | Gh_A11G0176-qR | GCCAGCCTCCTTCTCCTTTA |
| Gh_A11G0177 | Gh_A11G0177-qF | GGTTGTCACACCCGAAGAAC |
|  | Gh_A11G0177-qR | CATCCACCACCTTGAGTCCT |
| Gh_A11G0179 | Gh_A11G0179-qF | GCGCAAGCTCTCAATGTCAC |
|  | Gh_A11G0179-qR | TCCTCCTGGTGGGGATGTAG |
| Gh_A11G0184 | Gh_A11G0184-qF | TGGGTTCCATGTTCTGTCGG |
|  | Gh_A11G0184-qR | TCTGGTTGCCGAAGAGTGAC |
| GhHis3 | GhHis3-qF | TCAAGACTGATTTGCGTTTCCA |
|  | GhHis3-qR | GCGCAAAGGTTGGTGTCTTC |
