# Supplementary material for: A Genome Wide Association Study Revealed Key Single Nucleotide Polymorphisms/Genes Associated With Seed Germination in Gossypium hirsutum L
Source: Front Plant Sci. 2022 Mar 16;13:844946. doi: 10.3389/fpls.2022.844946 (PMC8967292; doi:10.3389/fpls.2022.844946)
Supplement: Supplementary file 5 [file Table_3.docx]

**Table S3.** The promising genes identified in multi-traits

| SNP | Gene ID | Homologue | Gene annotation | Traits |
| --- | --- | --- | --- | --- |
| A01_77149863, A01_77149864,  A01_77149876, A01_77149877,  A05:81655281, A08:314_10870, A08_53492125, A10_73015178, D13_18028416. | Gh_A01G1284 | AT3G61260 | Remorin family protein | GR  GI |
|  | Gh_A01G1285 | AT3G61260 | Remorin family protein |  |
|  | Gh_A08G0779 | [AT3G62310](https://www.arabidopsis.org/servlets/TairObject?type=locus&name=AT3G62310) | cilia- and flagella-associated protein 251-like |  |
|  | Gh_A08G0897 | [AT4G24060](https://www.arabidopsis.org/servlets/TairObject?type=locus&name=AT4G24060) | Dof-type zinc finger DNA-binding family protein |  |
|  | Gh_A10G1372 | [AT3G58690](https://www.arabidopsis.org/servlets/TairObject?type=locus&name=AT3G58690) | Protein kinase superfamily protein |  |
|  | Gh_D13G0891 | AT2G03830 | Root meristem growth factor |  |
|  | Gh_D13G0892 | [AT1G69030](https://www.arabidopsis.org/servlets/TairObject?type=locus&name=AT1G69030) | BSD domain-containing protein |  |
| A09_68240653, A09:68255852, A09_68257990, A09_68258389. | Gh_A09G1504 | [AT3G09600](https://www.arabidopsis.org/servlets/TairObject?type=locus&name=AT3G09600) | Homeodomain-like superfamily protein | GR  VI |
|  | Gh_A09G1508 | [AT2G29480](https://www.arabidopsis.org/servlets/TairObject?type=locus&name=AT2G29480) | Glutathione S-transferase TAU 8 |  |
|  | Gh_A09G1509 | [AT2G29420](https://www.arabidopsis.org/servlets/TairObject?type=locus&name=AT2G29420) | Glutathione S-transferase TAU 8 |  |
|  | Gh_A09G1510 | [AT3G09270](https://www.arabidopsis.org/servlets/TairObject?type=locus&name=AT3G09270) | Glutathione S-transferase TAU 8 |  |
|  | Gh_A09G1473 | [AT2G37540](https://www.arabidopsis.org/servlets/TairObject?type=locus&name=AT2G37540) | NAD(P)-binding Rossmann-fold superfamily protein |  |
